# Relationship between Photoelasticity of Polyurethane and Dielectric Anisotropy of Diisocyanate, and Application of High-Photoelasticity Polyurethane to Tactile Sensor for Robot Hands

**DOI:** 10.3390/polym13010143

**Published:** 2020-12-31

**Authors:** Masahiko Mitsuzuka, Yuho Kinbara, Mizuki Fukuhara, Maki Nakahara, Takashi Nakano, Jun Takarada, Zhongkui Wang, Yoshiki Mori, Masakazu Kageoka, Tsutomu Tawa, Sadao Kawamura, Yoshiro Tajitsu

**Affiliations:** 1 Research Organization of Science and Technology, Ritsumeikan University, Kusatsu, Shiga 525-8577, Japan; m-masa@fc.ritsumei.ac.jp (M.M.); wangzk@fc.ritsumei.ac.jp (Z.W.); 2 Mitsui Chemicals, Inc., Tokyo 105-7122, Japan; Yuho.Kinbara@mitsuichemicals.com (Y.K.); Maki.Nakahara@mitsuichemicals.com (M.N.); Takashi.Nakano@mitsuichemicals.com (T.N.); Masakazu.Kageoka@mitsuichemicals.com (M.K.); Tsutomu.Tawa@mitsuichemicals.com (T.T.); 3 Department of Robotics, Ritsumeikan University, Kusatsu, Shiga 525-8577, Japan; rr0079hi@ed.ritsumei.ac.jp (M.F.); rr0037ie@ed.ritsumei.ac.jp (Y.M.); Kawamura@se.ritsumei.ac.jp (S.K.); 4 Electrical Engineering Department, Graduate School of Science and Engineering, Kansai University, Suita, Osaka 564-8680, Japan; takarada@kansai-u.ac.jp

**Keywords:** photoelasticity, polyurethane, dielectric anisotropy, tactile sensor

## Abstract

Eight types of polyurethane were synthesized using seven types of diisocyanate. It was found that the elasto-optical constant depends on the concentration of diisocyanate groups in a unit volume of a polymer and the magnitude of anisotropy of the dielectric constant of diisocyanate groups. It was also found that incident light scattered when bending stress was generated inside photoelastic polyurethanes. A high sensitive tactile sensor for robot hands was devised using one of the developed polyurethanes with high photoelasticity.

## 1. Introduction

### 1.1. History and Purpose of This Study

Thus far, various methods of detecting stress inside a polymer based on the birefringence of light have been studied. For example, methods of irradiating a flexible and transparent polymer sheet with circularly polarized light and observing the fringes in the transmitted light through a polarizing filter [1,2,3,4,5,6], and methods of measuring changes in refractive index by stretching the polymer [7,8] have been studied. A tactile sensor [9] for stress detection based on the polarized light and the photoelasticity of transparent, flexible, and robust polyurethane is expected to realize a highly sensitive robot hand tactile sensor that imitates the tactile sensation of a human finger, but the performance of the sensor has not yet been fully verified.

The purpose of this research was to develop a highly sensitive tactile sensor of a robot hand and use it to grasp and process lightweight, soft and brittle objects such as foodstuff. It is necessary to develop a tactile sensor that can accurately measure a load of 0.1 N or less.

The goal of our research is to construct a versatile gripping system [10,11] at a low cost using a commercially available robot hand. Therefore, a cushioning function is also required so that a soft object is not crushed when it is gripped. Most commercially available robot hand opening/closing operation systems do not have the function of feeding back the force signal measured by the tactile sensor during operation. Therefore, a method of controlling the gripping force using the sensor when there is no feedback from the sensor to the control circuit of the hand will be described in the following example. The control program of a two-finger hand is set so that the closing distance between both fingers is 0.1 mm in each operation. Each time the finger closing operation is completed, the robot’s personal computer reads the gripping force signal from the tactile sensor, and if the target gripping force has not yet been reached, each finger is further closed by 0.1 mm. The operation of closing the hand is intermittently repeated several times until the target gripping force is reached, and when the gripping force reaches the target value, the finger closing operation is terminated.

However, for this purpose, the tactile sensor itself must have a cushioning function. That is, the gripping surface of the sensor must be deformed by up to 0.1 mm after the gripping surface comes in contact with the object. This function can reduce the force exerted on a soft and brittle object to avoid crushing it.

On the other hand, although polyurethane has been shown to exhibit photoelasticity in previous studies, the mechanism by which it exhibits photoelasticity and the relationship between the specific composition of polyurethane and the magnitude of photoelasticity have not yet been clarified. In this paper, we first propose the mechanism by which polyurethane exhibits photoelasticity, then present experimental results that support the validity of this mechanism. Then, a polyurethane with a composition suitable for the sensor is discussed. Finally, we show an example of a tactile sensor fabricated using the polyurethane obtained in this study.

In addition, for hydrogels [7], polybutadiene [8], acrylic copolymers [12], nylon [13,14], and gelatin gels [15], the magnitude of their birefringence is proportional to the strain. The birefringence mechanism of these polymers is also considered to be the same as that of polyurethane.

### 1.2. Mechanism Undelying Photoelasticity

**Scheme 1:**
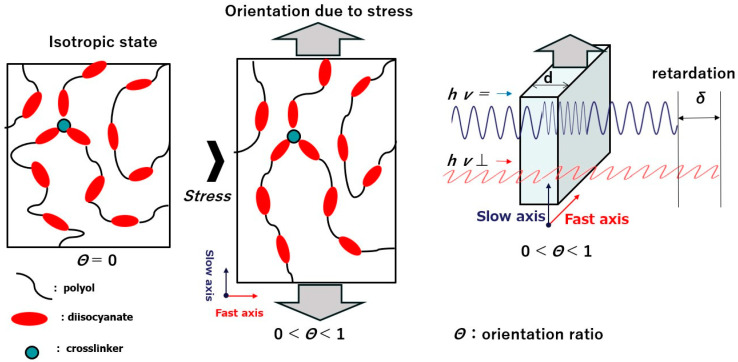
Mechanism underlying photoelasticity in polyurethane.

Scheme 1 shows how a polyurethane sheet produces photoelasticity when subjected to a tensile force. As shown in this figure, polyurethane forms a network by polymerizing a bifunctional polyol, a bifunctional diisocyanate, and a small amount of trifunctional triol as a crosslinker [16]. The diisocyanate is bound to the polyol along the long axis of its molecule, and when the sheet is stretched in the vertical direction, the long axis of the diisocyanate is partially oriented in the vertical direction.

Photoelasticity is a property in which a change in an optical parameter (refractive index) is caused by elastic deformation [17,18,19,20]. There are two constants: a photoelastic constant (**P**) and an elasto-optical constant (**E**). **E** is the magnitude of retardation in a sample with unit strain and unit thickness. Therefore, **E** is not directly related to the modulus of the polymer. To obtain the **E** of the polymer, a strain ε is applied by stretching a sheet with a thickness **d**, and the retardation (**δ**) between hν_⊥_ and hν = at that time is measured. Details are explained in Appendix A.

On the other hand, **P** is a function of **E** and **Y**. Therefore, the following relationship holds between **E** and **P**:

**P = E/Y** (1)

Assuming that the thickness of the film is d, the retardation is δ, and the stress applied to the film is **p**, the photoelastic constant (**P**) is expressed by:

**δ/d = P × p** (2)

A force sensor can be fabricated on the basis of this principle. An example of the configuration of such a sensor is shown in Appendix B [2,9].

**Scheme 2:**
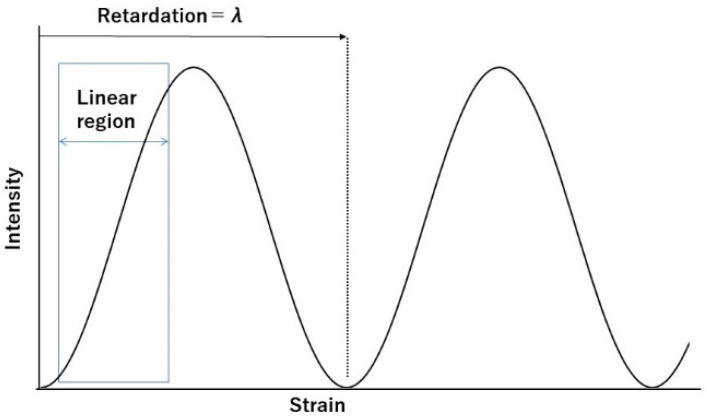
Relationship between strain and detected light intensity.

The relationship between the detected light intensity and the strain generated in the sheet is shown in Scheme 2 [9]. The light intensity increases with the strain, but when a certain strain is reached, the light intensity decreases thereafter, and the intensity becomes 0 when the retardation becomes equal to the light wavelength. As the strain further increases, the light intensity increases again: thus, the light intensity oscillates periodically as a function of strain. There is a region where the light intensity is approximately proportional to the strain; in this region, the light intensity and the force applied to the polymer have a linear relationship. A force sensor can be fabricated on the basis of the linear region in Scheme 2, but the limited range of measurable load is a disadvantage of this type of sensor.

The explanation thus far has been about applying a tensile force to a polymer, but when a compressive force is applied to a polymer, there is a proportional relationship between the force and the retardation, as in the case of a tensile force [8]. Thus, even when a compressive force acts on a polymer, the force applied to the polymer can be measured from the emitted light intensity on the basis of the same principle.

**Scheme 3:**
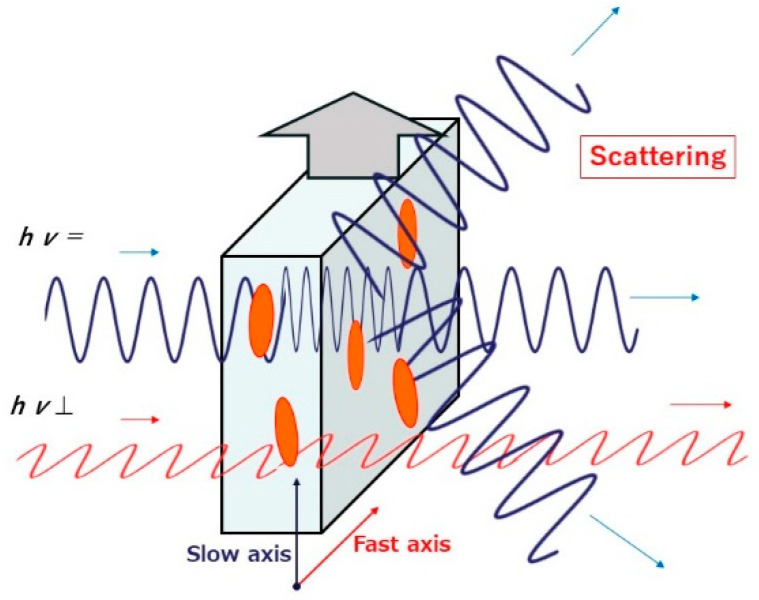
Light scattering in polyurethane.

We observed a phenomenon in which light scattered when stress was generated inside photoelastic polyurethanes [21,22,23,24] (Scheme 3). Specifically, a strand of polyurethane with a circular cross section was prepared, LED light of 630 nm wavelength was incident from one end, and the intensity of the light emitted from the other end was detected by a photodiode. It was found that as the bending angle of the strand increased, the emitted light intensity decreased [25]. This phenomenon has been used to realize pneumatically-driven flexible finger angle control for a robot finger [26]. From these results, it was considered that the same light scattering phenomenon would occur in a sheet of photoelastic polyurethane.

### 1.3. Previous Study of Photoelastic Polyurethane

**Figure 1:**
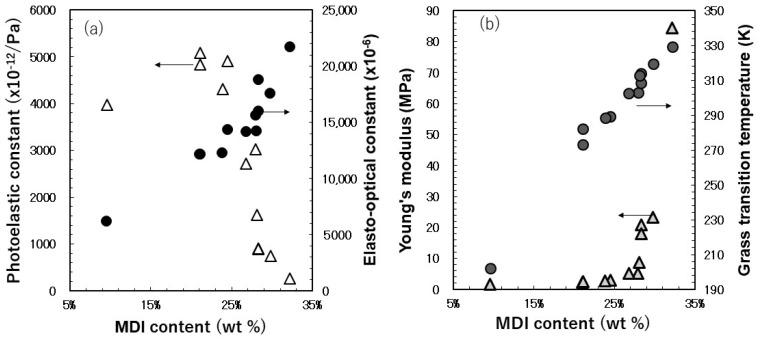
Photoelasticity measurement results of past research: (**a**) relationship between diisocyanate content and photoelasticity, and (**b**) relationships between diisocyanate content and elastic modulus and Tg.

It is known that the photoelasticity of polyurethane is derived from aromatic diisocyanate [16]. We assumed that the photoelasticity of polyurethane depended on the concentration of the aromatic ring of diisocyanate, and in an unpublished study, we investigated the relationship between the 4,4′-methylenediphenyl diisocyanate (MDI) content in polyurethane and photoelasticity using polybutadiene polyol and MDI (Figure 1).

The elasto-optical constant of polyurethane increased with the MDI content, but the photoelastic constant decreased when the MDI content exceeded 25 wt%. When the MDI content exceeded 25 wt%, the glass transition temperature (Tg) of polyurethane exceeded room temperature; thus, the Young’s modulus of the polyurethane increased, and the polyurethane became hard and difficult to deform. In Equation (1), the photoelastic constant (**P**) decreases as the Young’s modulus (**Y**) increases.

It was clear that the sensitivity of the sensor was increased by using a material with a large **P.** We therefore had to find a material with a large **E** and a **T_g_** that was considerably below the operating temperature of the sensor.

**Scheme 4:**
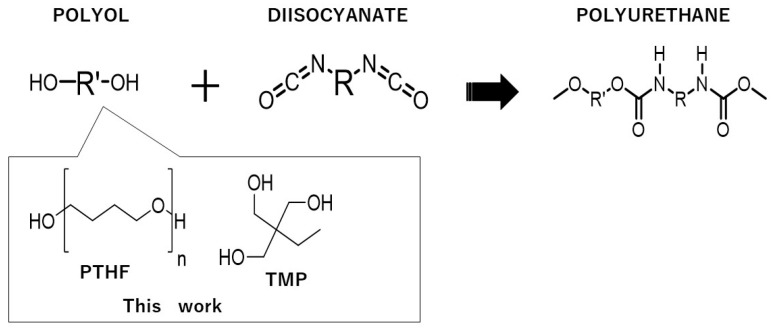
Reaction equation of the polyurethane that is the subject of this work.

As a result of investigating various polyols as the component of polyurethane, we decided to use polytetrahydrofuran (PTHF) for this study [25]. Scheme 4 shows the reaction equation of the polyurethane synthesized in this study. The polyol components are PTHF, which has a molecular weight of about 1000, and a trifunctional trimethylolpropane (TMP) as a crosslinker.

## 2. Experimental Section

### 2.1. Polyurethane Synthesis

#### 2.1.1. Crude Materials

Table 1 shows the abbreviations, chemical names, and suppliers of the reagents used.

#### 2.1.2. Preparation of Polyurethanes

Table 2 shows the raw material composition and R_iso_, which is the weight fraction of diisocyanate of eight types of polyurethane. A sample name with the prefix AR- indicates that an aromatic diisocyanate was used, and a sample with the prefix AL- indicates that an aliphatic diisocyanate was used. AR-2 was synthesized using two diisocyanates, TODI and MDI, with a weight ratio of about 30:70, respectively. The polymerization procedure of the main samples is described below.

##### 2.1.2.1. AR-2 Synthesis

A total of 2.0 g of antioxidant Irganox 245 was added to 1000 g of PTHF (hydroxyl value: 107.6 mgKOH/g) in a vessel, and the mixture was stirred at 393 K for 1 h under a reduced pressure of 0.8 kPa. After filling the inside of the vessel with nitrogen gas, the temperature was lowered to 353 K, 92.0 g of TODI was added to the vessel, and then the reaction was carried out at 363 K in nitrogen atmosphere. FT-IR measurement by an ATR-IR (JASCO Corporation FT/IR-4000) was used to examine the reaction, and the disappearance of isocyanate NCO vibration peak 2278 cm^−1^ was confirmed. A total of 10.0 g of TMP was added to the vessel, and the mixture was stirred under a reduced pressure until the TMP was completely dissolved. The product obtained was named prepolymer A and stored in a nitrogen atmosphere.

Subsequently, 100.0 g of prepolymer A was placed in a vessel and heated to 353 K. 0.23 g of the antifoaming agent BYK-088 and 17.6 g of MDI dissolved at 353 K were added, and the mixture was stirred for 1 min, defoamed under a reduced pressure for 1 min and poured into a mold. The mold was cured at 353 K for 18 h in a nitrogen-rich atmosphere, cooled to room temperature, and demolded to obtain a polyurethane sheet with a thickness of 4 or 2 mm.

##### 2.1.2.2. AR-1 and AR-3 Synthesis

A total of 2.0 g of Irganox 245 was added to 1000 g of PTHF in a vessel, and the mixture was stirred at 393 K under a reduced pressure for 1 h. After filling the inside of the vessel with nitrogen gas, the temperature of the vessel was lowered to 353 K, 10.0 g of TMP was added to the vessel, and the mixture was stirred under a reduced pressure until the TMP was completely dissolved. The mixture obtained was stored in a nitrogen atmosphere and is referred to as premix B.

Subsequently, 100.0 g of premix B was placed in a vessel and heated to 393 K (when using TODI for AR-1) or 353 K (when using MDI for AR-3). Then, 0.23 g of BYK-088 was added, followed by 29.4 g of TODI dissolved at 393 K or 27.8 g of MDI dissolved at 353 K, and the mixture was stirred, defoamed, poured into a mold, and cured as described in AR-2 Synthesis.

##### 2.1.2.3. AL-1 Synthesis

As described in Section 2.1.2.2, Irganox 245 was added to 1000 g of PTHF, dried in a vacuum and then 10.0 g of TMP was dissolved. Next, 0.2 g of dibutyltin dilaurate (DBTDL) was added as a catalyst and the mixture was stirred. This was stored in a nitrogen atmosphere and is referred to as premix C.

Subsequently, 100.0 g of premix C was placed in a vessel and heated to 353 K. Then, 0.23 g of BYK-088 was added, and the mixture was stirred and defoamed under a reduced pressure for 1 min. A total of 29.2 g of dicyclohexylmethane 4,4′-diisocyanate (H12-MDI) was added, and the mixture was stirred for 1 min, defoamed, poured into a mold, and cured as described in AR-2 Synthesis.

The other polyurethanes were also polymerized in the same manner. In all of the syntheses, the amount of diisocyanate was adjusted so that the molecular ratio [NCO]/[OH] was 1.05 (mol/mol). The number of moles of the NCO group was increased by 5% from the chemical equivalent used to suppress the effect of moisture in the raw material and in the atmosphere used for polymerization.

### 2.2. Method of Polyurethane Evaluation

#### 2.2.1. Photoelasticity Measurement Method

The photoelastic constants, elasto-optical constants, and Young’s modulus were measured by conventional methods [27,28,29]. Details are described in Appendix C. Regarding complex photoelasticity, it was measured while periodically changing the stress applied to the sample.

#### 2.2.2. Measurement of Glass Transition Temperature

The glass transition temperature (Tg) was determined by dynamic viscoelasticity measurement at a frequency of 10 Hz and a heating rate of 5 K/s. The temperature at which tan δ is maximized was defined as the glass transition temperature [30,31,32].

#### 2.2.3. Density Measurement

A density gradient tube was used to measure the density of polyurethane.

### 2.3. Calculation of Polarizability of Polyurethanes

#### 2.3.1. Molecular Structure Used in Calculations and Structural Optimization

The anisotropy of the polarizability of the diisocyanate group in polyurethane was calculated using the molecular structure in which the diisocyanate molecule at both ends was methoxylated. Before calculating the polarizability, the structure was optimized using the DFT method, B3LYP was used for the functional and 6–31 G (d, P) was used for the basis set [33]. Gaussian 09 Rev D.01 was used for the calculation.

#### 2.3.2. Polarizability Calculation and Definition of Anisotropy

In the calculation of polarizability [34,35,36,37], the straight line connecting the oxygen atoms of the methoxy group in the most stable structure was defined as the X-axis. The Y-axis was chosen in any direction orthogonal to the X-axis, and the Z-axis was chosen orthogonal to the X- and Y-axes. B3LYP was used for the functional and 6–31 + G (d, p) was used for the basis set [38].

Polarizability was determined for the 630 nm red light in the X-, Y- and Z-axis directions, and the anisotropy **α** was determined as:

**α** = **Px** − (**Py** + **Pz**)/2 (3)

where **Px**, and **Py**, and **Pz** are the polarizabilities along the X-, Y-, and Z-axes of the molecule, respectively. In this calculation, the diisocyanate molecule was approximated as a spheroid with the X-axis as the major axis. Since it is difficult to calculate the polarizability of the spheroid in the minor-axis direction, the mean of **Py** and **Pz** was regarded as the polarizability in the minor-axis direction.

### 2.4. Assembly of Bending Device

#### 2.4.1. Assembly of Bending Elasticity Measurement Device

**Figure 2:**
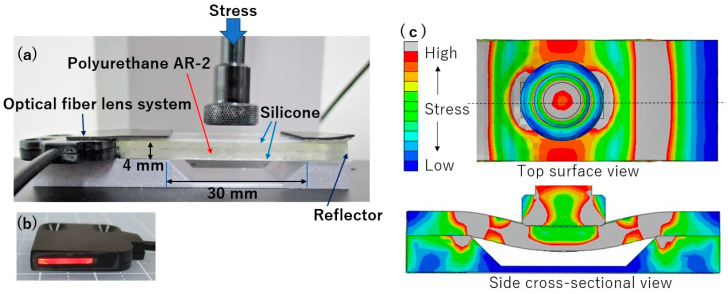
Bending elasticity measurement device and internal stress simulation (at 10 N load): (**a**) photographs of bending elasticity measurement device and (**b**) optical fiber lens head, and (**c**) simulation results of internal stress at 10 N load.

A bending elasticity measurement device was used for observing the phenomenon that when a load was applied to a photoelastic polyurethane sheet to generate bending elasticity, the light incident on the sheet was scattered by the birefringence generated in the polymer, causing light to leak from the sides and top surface (Figure 2).

**Figure 3:**
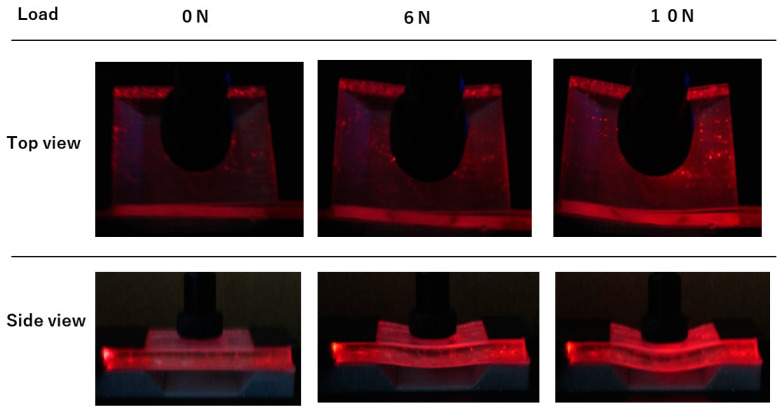
Photographs of light leakage due to bending.

The photoelastic polyurethane sheet had 0.1 mm-thick transparent silicone sheets fixed on both the upper and lower sides with an elastic adhesive. The polyurethane sheet was an AR-2 sheet with a thickness of 4 mm, a width of 2 cm, and a length of 5 cm. Both ends of the sheet were adhered to a support base, and a pushing load was measured at a constant speed using a disk-like probe with a flat contact surface and a diameter of 16 mm. Light with a wavelength of 630 nm was guided from one end face by a KEYENCE’s FU-12 optical fiber, and the light was focused into a parallel beam by a lens and was incident on the sheet. A reflective aluminum-deposited film was attached to the opposite end face of the polyurethane sheet. The light that leaked from the top and sides of the sheet was photographed with a digital camera while changing the load (Figure 3).

#### 2.4.2. Assembly of Light-Leakage-Type Load Sensor

**Figure 4:**
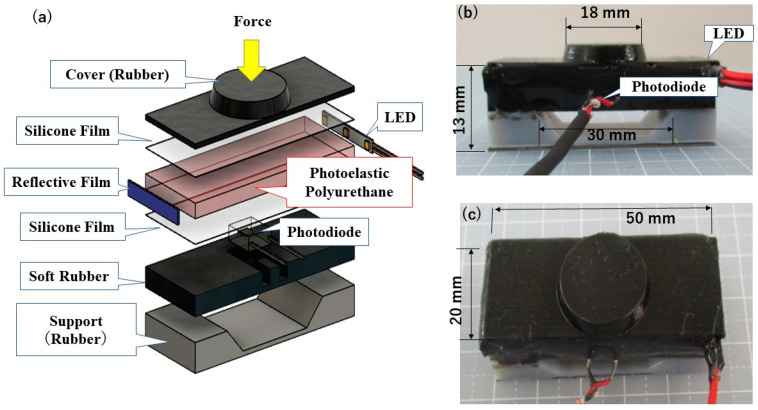
Structure of load sensor functioning on the basis of light leakage due to bending: (**a**) structural drawing, (**b**) side view of sensor, and (**c**) top view of sensor.

As shown in Figure 4, 0.1 mm-thick silicone sheets were fixed to both the upper and lower sides of an AR-2 polyurethane sheet with a transparent elastic adhesive (Semedine Super X Hyper Wide Clear). The refractive index of the polyurethane was 1.506, and that of the silicone was approximately 1.40 [39]. Owing to the refractive index difference of about 0.1, the light incident on the polyurethane/silicone interface at an angle close to parallel to the polyurethane sheet was totally reflected at the interface. Only the light that had entered the polyurethane sheet surface or polyurethane/silicone interface at an angle close to the vertical direction leaked out of the polyurethane sheet.

A 1 mm-thick flexible cover prepared by 3D printing using a Stratasys Tango Black material was placed on this sheet. At the center of this cover, there was a convex portion with a diameter of 18 mm and a height of 3 mm. The cover had the roles of gripping the object and blocking external light.

Under the polyurethane sheet, there was a 4 mm-thick rubber sheet prepared by 3D printing using a Stratasys Tango Black Plus material. There was a dent on the top of the rubber sheet into which the photodiode was inserted. The light-receiving surface of the photodiode was adhered to the silicone film using a transparent elastic adhesive. This rubber sheet had the roles of protecting the photodiode and blocking external light.

All of these parts were glued onto a support member prepared by 3D printing using a Stratasys Tango Gray material. There was a recess with a width of 30 mm and a depth of 5 mm at the center of the support base, and the polyurethane sheet bent downward when a load was applied. In this structure, birefringence occurred inside the polyurethane, and the light of the white LED incident from the side surface of the sheet scattered outward. Part of this scattered light was detected by a planar photodiode (Hamamatsu Photonics S2506-02) that was sensitive to visible light. The sides of the polyurethane sheet were coated with a black elastic adhesive (Cemedine Super X Black) to block light. In a transparent resin, the shorter the light wavelength, the greater the birefringence tends to be [40], so it is presumed that white light had a higher scattering intensity than red light.

The photocurrent from the photodiode was amplified in the first stage by a common current–voltage conversion circuit [9] using an operational amplifier (Texas Instruments LMC6482). The output voltage was shifted [41] by a differential amplifier circuit so that the output voltage without a load became 0 V. At the same time, the second stage amplification was performed and the amplified photocurrent was recorded with a KEYENCE digital recorder (KEYENCE NR-600/NR-TH08). A second-order passive low-pass filter with a cutoff frequency of 16 Hz was incorporated into the circuit to reduce the noise with a frequency of 60 Hz from the commercial power supply.

#### 2.4.3. Constant-Speed Loading Test of Sensors

An IMADA ZTA-50N force gauge was attached to an IMADA EMX measuring stand. A disk-shaped probe with a diameter of 16 mm was attached to the tip of the force gauge, and the force gauge was pressed vertically from above against the gripping surface of the sensor at a descent speed of 0.5 mm/min using a measuring stand. The displacement, stress, and sensor output were recorded with the KEYENCE digital recorder while applying a load to the top surface of the sensor.

## 3. Results and Discussion

### 3.1. Characterization of Polyurethanes

#### 3.1.1. Photoelasticity and Young’s Modulus

Table 3 shows the photoelastic constant, elasto-optical constants, and Young’s modulus obtained from the measurement of photoelasticity using a polyurethane sheet with a thickness of 2 mm.

#### 3.1.2. Dynamic Viscoelasticity Measurement

**Figure 5:**
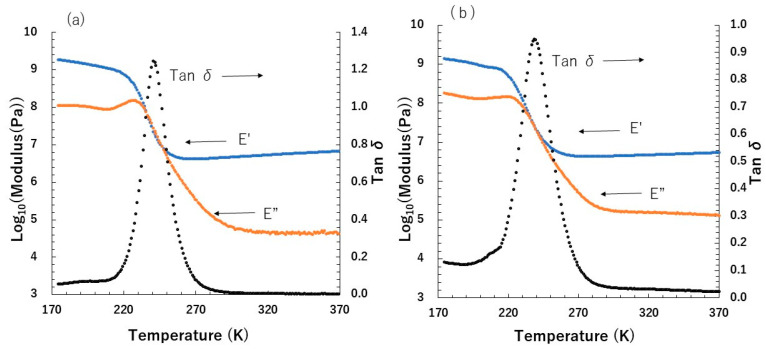
Dynamic viscoelasticity measurement results of polyurethanes: (**a**) AR-2 and (**b**) AL-1 samples.

Figure 5 shows the results of dynamic viscoelasticity measurements of AR-2 and AL-1. The **T_g_** values of all samples are also listed in Table 3. All eight types of polyurethane had a **T_g_** of less than 242 K and remained flexible even at temperatures below 273 K.

**Figure 6:**
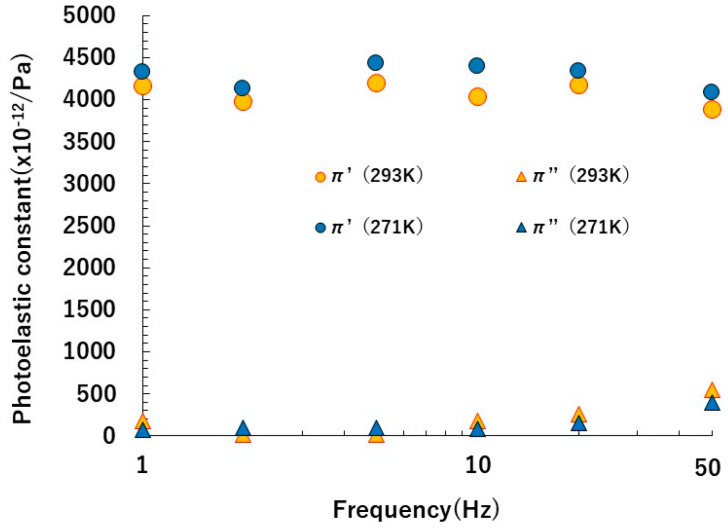
Dynamic photoelasticity measurement results of AR-2.

Polyurethanes prepared from PTHF and diisocyanate have been studied, but most of them contained short-chain diols (e.g., 1,4-butanediol) as raw materials [31,32,42,43]. However, the polyurethanes synthesized in this study did not contain a short-chain diol. In Figure 6, the peak of tan δ was single, and this peak indicated the temperature at which the soft segment of the polyurethanes changed from the glassy state to the elastic amorphous state.

#### 3.1.3. Complex Photoelasticity

Figure 6 shows the results of the complex photoelasticity measurement of AR-2 at 293 and 271 K. From the measurement of the complex photoelasticity, the real component **π′** showed a high photoelastic constant that remained the same even at 271 K up to a frequency of 50 Hz, whereas the imaginary component **π″** was almost two orders of magnitude lower than **π′**. When this polyurethane was applied to a sensor, the sensitivity was constant as a function of the frequency even at a low temperature of 271 K, and a rapid response could be expected.

#### 3.1.4. Polarizability Anisotropy of Diisocyanate and Origin of Photoelasticity

**Scheme 5:**
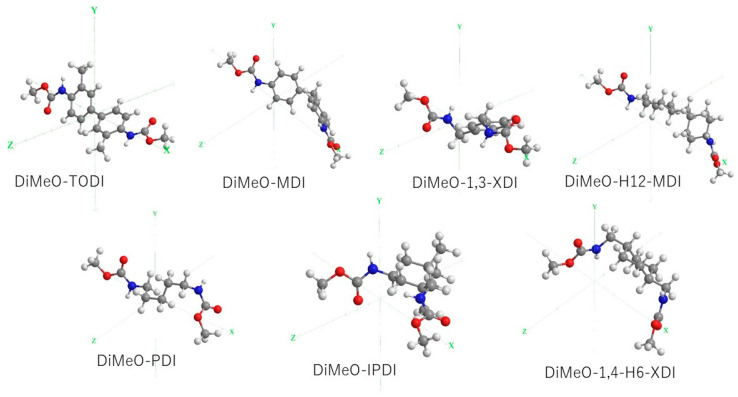
Stable structures of the molecules used to calculate the polarizability.

The cause of the birefringence when the polymer sheet was stretched was the orientation of the diisocyanate group in the tensile direction. As shown in Scheme 1, the diisocyanate groups in the polymer chain are oriented in the tensile direction from the random state, and the strength of the orientation θ is proportional to the magnitude of the strain ε of the sheet [44,45]. The diisocyanate groups have polarizability anisotropy, and the anisotropy could be approximated by a spheroid [44] with a polarizability whose major axis is the X-axis connecting two isocyanate groups. Therefore, the polarizability in the minor-axis direction of the spheroid was approximated from the averages of the polarizabilities **Py** and **Pz**, and the anisotropy was calculated using Equation (3). The stable structures of the dimethoxy-diisocyanate molecules used in the calculation and the X-, Y-, and Z-axes of the molecules are shown in Scheme 5.

The calculated results of anisotropies are shown in Table 4. Note that in the calculation of the polarizabilities **Py** and **Pz** of TODI also shown in Table 4, the polarizabilities were calculated by rotating the Y- and Z-axes by 30° and by 60° around the X-axis, respectively. The calculated anisotropy did not change even when the Y- and Z-axes were rotated. From this result, it was considered that the method of calculating the anisotropy by selecting the Y-axis in any direction was appropriate.

Next, we consider the anisotropy that occurs in the polyurethane sheet per unit volume when the strain ε was applied. Using the anisotropy of a single diisocyanate group (**α**), the ease of orientation (**β**), the density of polyurethane (**D**), the weight ratio of diisocyanate in polyurethane (**R_iso_**), and the molecular weight of diisocyanate (**M**), we can write the elasto-optical constant **E** as:

**E** ∝ **α** × **β** × **D** × **R_iso_**/**M** (4)

where **β** is the ratio of **θ** to **ε** (**θ**/**ε**). Generally, **β** depends on the type of polyurethane, but assuming that polyurethanes with the same polyol have the same **β** value even when the diisocyanate is different, **β** is a constant within the scope of this study. Therefore, Equation (4) becomes:

**E** = **C** × **α** × **D** × **R_iso_**/**M** (5)

where **C** is a constant obtained in the experiment. The **d**, **R_iso_**, **M**, and **α** of each polyurethane and the calculated **α** × **β** × **D** × **R_iso_**/**M** are shown in Table 5 together with **E**.

**Figure 7:**
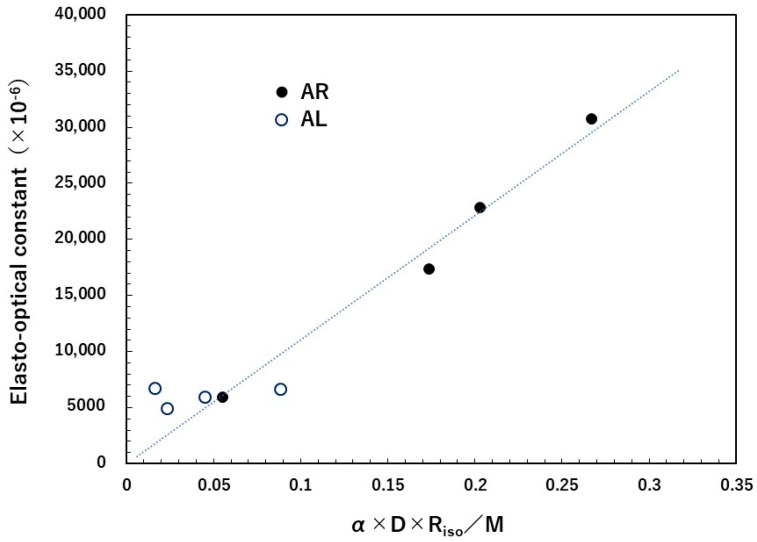
Relationship between **α** × **D** × **R_iso_**/**M** and the elasto-optical constant of polyurethanes.

In Figure 7, the plot of the polyurethane prepared using an aromatic diisocyanate was close to a straight line passing through the origin. The constant **C** in Equation (5) was calculated from this plot to be 110,000. This indicated that the anisotropy of the polarizability of diisocyanate was the origin of the photoelasticity of polyurethane. On the other hand, the plot of the polyurethane prepared using aliphatic diisocyanate did not show this tendency, but it was presumed that since the polarizability of the aliphatic diisocyanate was small, the accuracy of **α** was low.

### 3.2. Characterization of Sensor

#### 3.2.1. Observation of Light Leakage and Finite Element Simulation

As shown in Figure 2a, the displacement–stress characteristic was measured while pushing down on the center of the AR-2 polyurethane sheet with a disk-shaped probe at a speed of 0.5 mm/min, where the displacement was 2.33 mm when the load was 10 N. On the other hand, to capture the stress distribution upon loading, a finite element simulation was conducted using Abaqus software (SIMULIA, Dassault System, MA). The Young’s modulus of the AR-2 polyurethane sheet was set to 4.7 MPa as obtained in measurements (Table 3). Simulation results yielded a 10.0 N force at a displacement of 2.34 mm, which is in good agreement with the measurement results. Figure 2c shows the stress distribution on the surface and cross section of the polyurethane sheet at a load of 10 N. The areas with the highest stress are shown in gray.

After darkening the laboratory, the red LED of the optical fiber lens system (Figure 2b) was turned on, a load was applied, and the sheet was photographed from the top or side of the sheet with a digital camera at an exposure time of 1 s. Light leaked from the top and sides of the sheet when the load was applied, and as the load increased, the intensity of the leaked light increased (Figure 3). From the simulation results in Figure 2c, there was a high-stress area at the bottom of the sheet, and it was probable that the LED light scattered in this stressed area leaked from the side and top surfaces of the sheet. This means that the incident light from the LED strongly scattered owing to the anisotropy of the polarizability of the polyurethane induced by stress. It is considered that the stress inside polyurethane can be measured by measuring the intensity of this scattered light.

#### 3.2.2. Light-Leakage-Type Load Sensor

The structure of a tactile sensor is shown in Figure 4. In the following experiments, an AR-2 polyurethane sheet of 2 mm thickness was used. The key feature of this sensor was that the polyurethane sheet itself bends and acts as a cushion.

**Figure 8:**
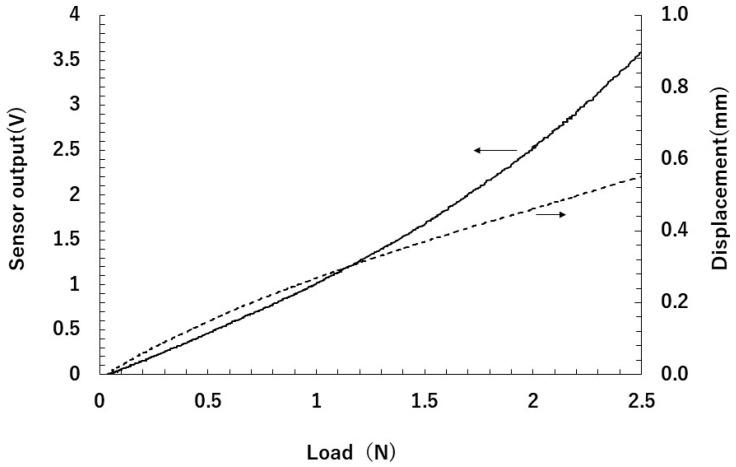
Results of constant-speed loading test (at 0.5 mm/min).

The force curve obtained in the constant-speed load test is shown in Figure 8. Up to a load of 1 N, the output of the sensor was almost proportional to the load. The load was 0.3 N at the displacement of 0.1 mm; thus, the cushioning function of the sensor was confirmed.

**Figure 9:**
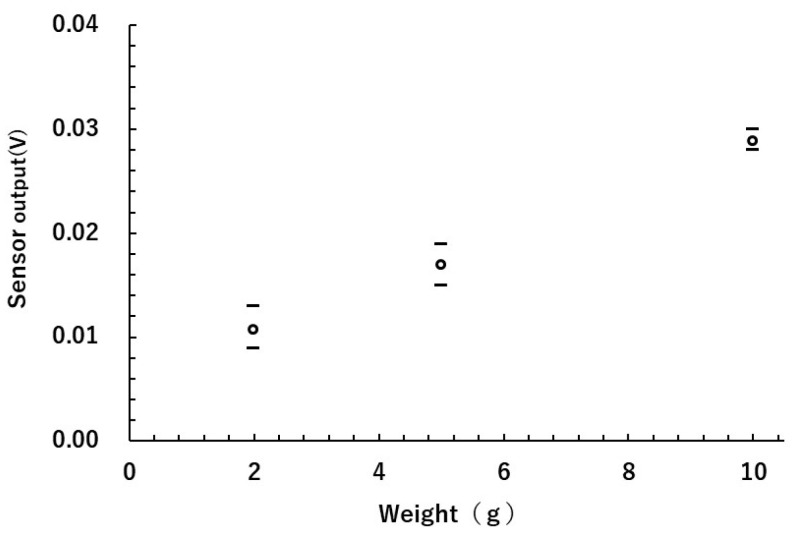
Output of a sensor when measuring using standard weights. Open circles are averages of 10 measurements and horizontal bars show the maximum and minimum values.

A standard test weight was placed on the convex part of the sensor and the sensor output was measured. Figure 9 shows the results of 10 measurements using the same weight. The averages of the 10 measurements are indicated by open circles, and the maximum and minimum values are indicated by horizonal bars.

The relationship between the load and the sensor output was almost linear from 2 to 10 g. The average of the 10 measurements with a weight of 10 g was 0.0288 and the standard deviation was 0.00060; thus, the coefficient of variation was 0.021, indicating a sufficiently accurate measurement.

### 3.3. Comparison with Other Grip Sensors

Since the 2000s, research has been conducted on grip sensors that imitate the shape of human fingers by embedding sensors such as strain gauges inside silicone resin [46,47,48,49]. A uSkin [50] could detect a force of 0.25 N, but its sensitivity was still insufficient for our purposes. Capacitive sensors [51] were also commonly used to detect force, but their structure is complex. A soft tactile sensor [52] could be expected to have a sensitivity of 0.25 N or less, but the process of manufacturing such devices is complex. A piezoresistive sensor [53] could measure a force of 0.1 N with high sensitivity, but the structure of the sensor is still complex.

On the other hand, the light-leakage-type load sensor obtained in this study could detect a minimum load of 2 g (0.02 N), and a load of 10 g (0.098 N) could be measured with high accuracy. In addition, the structure of this sensor is easy to manufacture because it is prepared by laminating rubber sheets. Mass-produced LEDs and photodiodes are used, and the detection circuit is also prepared by a general operational amplifier; this sensor could be produced at a low cost. Additionally, this sensor has the cushioning function, so it could be attached directly to a commercially available robot hand.

## 4. Conclusions

In this work, eight types of polyurethane were synthesized using seven types of diisocyanate. It was found that the elasto-optical constant (**E**) depends on the concentration of diisocyanate groups in a unit volume of a polyurethane and the magnitude of anisotropy of the dielectric constant of diidocyanate groups (**α**):

**E** = **C** × **α** × **D** × **R_iso_**/**M** (6)

here, **D** is the density of polyurethane, **R_iso_** is the weight fraction of diisocyanate, **M** is the molecular weight of diisocyanate, and **C** is a constant obtained experimentally.

It was also found that incident light scattered when stress was generated inside photoelastic polyurethanes. In addition, a two-finger robot hand tactile sensor was fabricated using AR-2 polyurethanes with high photoelasticity obtained in this study and its characteristics were evaluated. This sensor bended under load and could measure the load by measuring the intensity of scattered light. This sensor was highly sensitive and could detect a load of 2 g or more and accurately measure a load of 10 g.

This sensor also had the cushioning function; thus, it can be attached directly to a commercially available robot hand. In addition, this sensor was easy to manufacture, so this study was in line with our development goals.

## Figures and Tables

**Table 1:** List of raw materials used in this work.

Abbreviation	Chemical Name	CAS No.	Supplier
TODI	3,3’-Dimethyl-4,4’-biphenylene diisocyanate	91-97-4	Nippon Soda
MDI	4,4’-methylenediphenyl diisocyanate	101-68-8	Mitsui Chemicals
1,3-XDI	1,3-Bis(isocyanatomethyl)benzene	3634-83-1	Mitsui Chemicals
H12-MDI	Dicyclohexylmethane 4,4’-diisocyanate	5124-30-1	Tokyo Chemical Industry
PDI	1,5-Diisocyanatopentane	4538-42-5	Mitsui Chemicals
IPDI	Isophorone diisocyanate	4098-71-9	Tokyo Chemical Industry
1,4-H6-XDI	1,4-bis(isocyanatomethyl)cyclohexane	10347-54-3	Mitsui Chemicals
DBTDL	Dibutyltin dilaurate	77-58-7	Fujifilm Wako Pure Chemical Corporation
PTHF	Poly(tetrahydrofuran)	25190-06-1	Hodogaya Chemical
TMP	2-(hydroxymethyl)-2-ethylpropane-1,3-diol	77-99-6	Tokyo Chemical Industry
BYK-088	—	—	BYK Japan
Irganox 245	Triethylene glycol bis(3-tert-butyl-4-hydroxy-5-methylphenyl)propionate	36443-68-2	BASF Japan

**Table 2:** Raw material composition of produced polyurethane.

Sample Name	AR-1	AR-2 *^1^	AR-3	AR-4	AL-1	AL-2	AL-3	AL-4
Diisocyanate name	TODI	TODI/MDI	MDI	1,3-XDI	H12-MDI	PDI	IPDI	1,4-H6-XDI
PTHF(g)	99							
TMP(g)	0.99							
Diisocyanate(g)	29.4	9.11/19.2	27.8	20.9	29.2	17.1	24.7	21.6
R_iso_	0.227	0.071/0.150	0.218	0.173	0.226	0.146	0.198	0.177

*^1^ Polyurethane made of TODI/MDI = 30/70 (weight/weight) mixture.

**Table 3:** Properties of synthetic polyurethane.

Sample Name	AR-1	AR-2	AR-3	AR-4	AL-1	AL-2	AL-3	AL-4
Type of diisocyanate	TODI	TODI/MDI	MDI	1,3-XDI	H12-MDI	PDI	IPDI	1,4-H6-XDI
Photoelastic constant (10^−12^/Pa)	10200	4860	3510	1650	1850	1380	1190	1410
Elasto-optical constant (10^−6^)	30700	22800	17300	5860	6620	5930	4870	6650
Young’s modulus (10^6^ Pa)	3.01	4.69	4.93	3.55	3.58	4.3	4.09	4.72
Glass transition temperature (K)	239.5	240.8	241.8	221.9	238.5	215.3	231.7	224.3

**Table 4:** Polarizability calculation results. The diisocyanates with both ends methoxylated were used in the polarizability calculation.

Diisocyanate	Polarizability in 630 nm Light (arb. unit)			(P_y_ + P_z)_/2	α
	P_X_	P_y_	P_z_		
TODI	488	218	171	194.5	293.5
		173 *^1^	217 *^1^	195	293
		149 *^2^	240 *^2^	194.5	293.5
MDI	382	197	191	194	188
H12-MDI	300	201	200	200.5	99.5
1,3-XDI	215	171	143	157	58
PDI	172	119	132	125.5	46.5
IPDI	216	213	167	190	26
1,4-H6XDI	184	178	155	166.5	17.5

*^1^ Y- or Z-axes was rotated 30 degrees around the X-axis. *^2^ Y- or Z-axes was rotated 60 degrees around the X-axis.

**Table 5:** List of densities, diisocyanate contents of polyurethanes, molecular weights, and polarizability anisotropies of the diisocyanates.

Sample Name	AR-1	AR-2	AR-3	AR-4	AL-1	AL-2	AL-3	AL-4
D (g/cm^3^)	1.06	1.063	1.063	1.048	1.033	1.028	1.028	1.034
R_iso_	0.227	0.22	0.218	0.173	0.226	0.146	0.198	0.177
M (g/mol)	264.3	–	250.3	188.2	262.4	154.2	222.3	194.2
α	293.5	–	188	58	99.5	46.5	26	17.5
α × D × R_iso_/M	0.2672	0.2033 *	0.174	0.0559	0.0885	0.0453	0.0238	0.0165
E (10^−6^)	30700	22800	17300	5860	6620	5930	4870	6650

*: The contribution from TODI is 0.0838 and that from MDI is 0.1195.

**Scheme A1:**
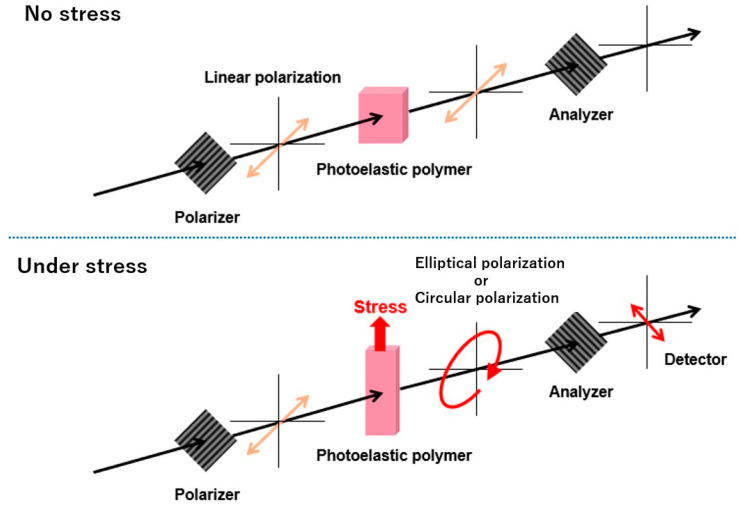
Stress measurement method using photoelasticity.

**Scheme A2:**
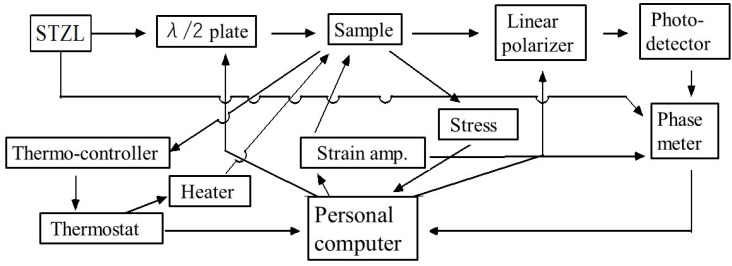
Photoelasticity measurement method.

## Data Availability

We comply with laws and other requirements.

## Appendix A

When the strain is minute, the degree of orientation (**θ**) is proportional to the strain (**ε**) in the vertical direction. Light is incident on the surface of the sheet in which the diisocyanate is oriented in the vertical direction. The velocity of the light whose electrical vector oscillates vertically (hν=) becomes low in the polymer, because the dielectric constant of the diisocyanate molecule is larger in the long-axis direction. On the other hand, the velocity of the light whose electrical vector vibrates in the horizontal direction (hν_⊥_) is relatively high, because the dielectric constant of the diisocyanate molecule is smaller in the minor-axis direction than in the major-axis direction. Therefore, hν = lags behind hν_⊥_ in the phase of the emitted light. This delay is called retardation (**δ**). Assuming that the thickness of the film is **d**, the Young’s modulus of the film is **Y**, and the stress **p** applied to the film, the elasto-optical constant (**E**) is expressed by:

**E = Y****×****δ/(d****×****p)** (A1)

The photoelastic constant (**P**) is represented by:

**P** = **δ**/(**d** × **p**) (A2)

Therefore, the following relationship holds between **E** and **P**:

**P****= E/****Y** (A3)

In the region where the strain ε is small, **p**, **Y**, and **ε** are related by:

**Y = p/ε** (A4)

Equation (A5) is derived from Equations (A4) and (A1):

**E = δ/(d****×****ε)** (A5)

Equation (A5) indicates that **E** is the magnitude of retardation in a sample with unit strain and unit thickness. Therefore, **E** is not directly related to the modulus of the polymer. To obtain the **E** of the polymer, a strain ε is applied by stretching a sheet with thickness **d**, and the retardation (**δ**) between hν_⊥_ and hν = at that time is measured.

By rewriting Equation (A2) as:

**δ/d = P****×****p** (A6)

we can see that the phase retardation of the sample with unit thickness (**δ**/**d**) is proportional to the constant **P** and **p**. Therefore, **p** can be obtained by measuring the **δ** of a material for which **P** is known.

## Appendix B

Two linear polarizing plates, a polarizer and an analyzer, are installed in front of and behind a polymer sheet. The polarization axis of the polarizer is tilted 45° from the vertical axis, and the polarization axes of the polarizer and analyzer are tilted 90° from each other. The plane of the electrical vector of light passing through the polarizer is tilted 45° from the vertical axis. If the polymer is not stressed, there is no retardation between the vertical and horizontal components of this light, and light cannot pass through the analyzer on the emitted light side. However, when a vertical force is applied to the polymer sheet, photoelasticity is generated inside the polymer and phase retardation is induced, resulting in elliptically polarized light. This elliptically polarized light contains components that pass through the analyzer on the emitted light side, and the force applied to the sheet can be measured by detecting the intensity of light that has passed through [2,9].

## Appendix C

Equation (A7) is the optical indicatrix of the crystal using orthogonal coordinates [27]:

(A7) $$\frac{x_{1}{}^{2}}{n_{1}{}^{2}}+\frac{x_{2}{}^{2}}{n_{2}{}^{2}}+\frac{x_{3}{}^{2}}{n_{3}{}^{2}}=1$$

here, **n***_i_* is the refractive index of the **x***_i_*-axis (*i* = 1, 2, 3). Under the application of a small stress **T**_3_ parallel to the **x**_3_-axis (principal axis), we derive Equation (A8) from Equation (A7):

**n_1_ = n_o_ − δn_1_** (A8)

**δn_1_ = 1/2 n_o_^3^π_31_T_3_** (A9)

here, ***π*_31_** is the photoelastic constant. The second term **δn**_1_ in Equation (A8), which is obtained by applying **T**_3,_ represents photoelasticity. The system developed here is based on the simultaneous measurement of small changes in retardation and stress under the application of a small strain. In this system, we adopted a measurement method based on the heterodyne interferometry principle to improve the accuracy of retardation measurement, the details of which were reported previously [28,29]. We only provide a brief outline as follows. A stabilized transverse Zeeman laser (STZL) emits orthogonally polarized two-frequency light of **λ** = 632.8 nm. A half-wave plate and a linear polarizer are set on rotating stages controlled by a microcomputer. The laser beam passes through the half-wave plate, the film sample, which undergoes intrinsic-birefringence-induced and stress-induced retardation, and the linear polarizer, which is incident to a photodetector. The optical beat signal obtained at the photodetector is synchronously detected by a phase meter. The output signals are stored in a personalcomputer (Scheme A2). Furthermore, in the measurement of complex photoelasticity, the photoelasticity is measured while periodically changing the stress applied to the sample.
